# The British Hospitals Association

**Published:** 1911-10-07

**Authors:** 


### THE BRITISH HOSPITALS ASSOCIATION.

### Important Meeting at Manchester.

### THE NATIONAL INSURANCE BILL.

### RESOLUTION ADOPTED CALLING FOR ITS AMENDMENT.

The Second Annual Conference of the British Hospitals Association was held in Manchester on September 28 and 29. By permission of the Lord Mayor of the city (Councillor Chas. Behrens), the meetings were held on the Lord Mayor's parlour of the Town Hall, and the Lord Mayor was himself present at the opening of the proceedings on the first day to welcome the Association in the name of the city.

Some two hundred representatives of voluntary hospitals in towns and districts throughout the country attended the Conference. By resolution of the Conference Sir William Cobbett, chairman of the Board of Management of the Manchester Royal Infirmary, was voted to the chair and at the platform end were the following: the honorary treasurers of the Association, Mr. Conrad W. Thies, Royal Free Hospital, London, and Mr. E. Stewart Johnson, of the Hospital for Sick Children, Great Ormond Street, London; the honorary secretaries, Dr. D. J. Mackintosh, M.V.O., Western Infirmary, Glasgow, and Mr. A. William West, St. George's Hospital, London; Members of the Council, Sir Henry Burdett, K.C.B.; Mr. Howard J. Collins, General Hospital, Birmingham; Dr. G. C. Friend, St. George's Hospital, London; Mr. L. W. Glenton-Kerr, Great Northern Hospital, London; Mr. Chas. Lupton, General Infirmary, Leeds; and Col. J. A. Roxburgh, Western Infirmary, Glasgow; the local honorary secretary, Mr. W. G. Carnt, Manchester Royal Infirmary. Amongst those who attended were:—

Miss Alexander, Royal Alexandra Infirmary, Paisley; Miss McCall Anderson, St. George's Hospital, S.W.; Rev. G. C. Aspinall, Royal Halifax Infirmary; Mr. F. G. Adnitt, Northampton General Hospital; Mr. A. W. Arning, Royal Eye Hospital.

Mr. Willoughby Bullock, Seaside Convalescent Hospital, Seaford, Sussex; Mr. W. G. Black, Glasgow Royal Infirmary and Glasgow Eye Infirmary; Mr. F. W. Beadon, Huddersfield Infirmary; Mr. J. J. Barron, Royal Infirmary, Bradford; Mr. F. J. Bray, General Infirmary, Leeds; Mr. J. C. Buchanan, Metropolitan Hospital, Kingsland Road, London, N.E.; Mr. T. F. Bache, West Bromwich and District Hospital; Mr. W. Bray, Workhouse Infirmary, Brownlow Hill, Liverpool; Mr. Benjamin Brooks, Hull Royal Infirmary; Mrs. A. Bayley, Northern Hospital, Manchester; Mr. W. Buckley, J.P., Manchester Children's Hospital; Mr. J. Bell, Royal Infirmary, Manchester; Mr. A. C. Bowdler, Blackburn Infirmary; Dr. Judson S. Bury, Manchester Royal Infirmary; Miss Broadbent, Manchester Ear Hospital; Mr. Fredk. Burton, Salford Royal Hospital; Mr. Samuel Baron, Ancoats Hospital.

Mr. A. H. Cottom, J.P., Blackburn and E. Lancs Infirmary; Rev. M. Campbell, Dumfries and Galloway Royal Infirmary; Captain G. R. Crosfield, Warrington Infirmary and Dispensary; Mr. J. Cooper, Royal Infirmary, Manchester; Mr. J. A. Cowley, Victoria Infirmary, Northwich; Mr. W. W. Cannon, Bolton Infirmary; Mr. Geo. C. Campion, Dental Hospital, Manchester; Dr. A. K. Chalmers, M.O.H., Glasgow; Mr. Neville Clegg and Mr. Brian Crossley, Royal Infirmary, Manchester; Mr. Edward P. Collett, Dental Hospital, Manchester; Mr. Alfred Crewdson and Dr. E. J. Carlisle, Hospital for Incurables, Mauldeth.

Mr. J. Dunn, Victoria Infirmary, Glasgow; Mr. C. R. Scrase Dickins, Royal Sussex and County Hospital, Brighton; Mr. J. Dendy, Manchester Children's Hospital; Mr. Louis M. Dick, Royal National Pension Fund; Rev. D. Dorrity, St. John's Hospital, Manchester (for the Ear and Eye); Mr. T. Drew, Royal Eye Hospital; Dr. Chas. Dreyfus, Jewish Hospital, Manchester; Dr. W. A. Donald, St. Mary's Hospital; Mr. Joseph Duckworth, Manchester Northern Hospital.

Mr. H. J. Eason, Manchester Children's Hospital; Mr. E. Entwistle, J.P., St. John's Hospital for Ear and Eye, Manchester.

Mr. A. Scott Finnie, Royal Infirmary, Aberdeen; Lieut.-Colonel Sir D. Fayrer, Bt., F.R.C.S., Royal Infirmary, Edinburgh; Mr. W. Frost, Macclesfield General Infirmary; Mr. Edgar Fitton, Dewsbury and District General Infirmary; Alderman J. Fildes, Manchester Consumption Hospital.

Rev. R. B. Gwydir, Swansea Eye Hospital; Mr. A. G. Griffiths, East Suffolk Hospital, Ipswich; Dr. T. Wardrop Griffith, Leeds General Infirmary; Mr. Robert Gourlay, LL.D., Glasgow Maternity and Women's Hospital; Mr. A. E. Gaddum, Ancoats Hospital.

Sir A. A. Haworth, Bart., M.P.; Mr. James Henderson, Jessop Hospital for Women, Sheffield; Mr. Edward Hemingway, Dewsbury and District General Infirmary; Mr. H. A. Heywood, Manchester Children's Hospital; Mr. C. W. Hunt, Manchester Consumption Hospital; Alderman Edward Holt, Manchester Northern Hospital; Mr. Chas. Hopkinson, Manchester Royal Infirmary; Dr. C. C. Heywood, Manchester Children's Hospital; Mr. E. J. Hoyle, Hospital for Incurables; Mr. John Hall and Mr. E. Hayward, Ancoats Hospital; Sir Alfd. Hopkinson.

Mr. H. Johnson, Leicester Infirmary; Mr. Thos. Jones, West Bromwich District Hospital.

Mr. L. H. Kerr, Great Northern Hospital, Holloway Road, London, N.; Mr. W. Kay, Bolton Infirmary; Mr. P. W. Kessler, Royal Eye Hospital, Manchester; Mr. H. Keeling, Birmingham Dental Hospital; Mr. E. A. Kelp, Manchester Ear Hospital; Mr. G. D. Kelley, Manchester Royal Infirmary; Rev. J. G. Kemp, St. John's Hospital for the Ear and Eye, Manchester.

Dr. G. R. Livingstone, Royal Infirmary, Dumfries and Galloway; Mr. W. F. Lister, Royal Infirmary, Doncaster; Mr. R. G. Lawson, Christie Cancer Hospital, Manchester; Mr. Levy Eliot, Manchester Royal Infirmary; Mr. J. I. Loewy, Jewish Hospital, Manchester; Mr. Geo. H. Leigh, Manchester Children's Hospital.

Dr. D. O. Macgregor, Victoria Infirmary, Glasgow; Mr. G. Martyr, Royal Victoria Hospital, Bournemouth; Mr. Wm. Moody, General Hospital, Altrincham; Mr. Thomas Maddock, Cottage Hospital, Wellingborough; Mr. Chas. A. Mason, Birmingham and Midland Eye Hospital, Church Street, Birmingham; Mr. Jas. Macfarlane, Glasgow Royal Infirmary; Mr. A. Hyslop Maxwell, Liverpool Infirmary for Sick Children; Dr. Wm. Milligan, Manchester Ear Hospital; Mr. Percy H. Marriott, Manchester Dental Hospital; Dr. C. H. Melland, Ancoats Hospital.

Mr. A. Naldrett, Royal Southern Hospital, Liverpool; Mr. Frederick Neden, County Hospital, York; Mr. J. S. Neil, General Hospital, Wolverhampton; Mr. J. Hall, Manchester Skin Hospital; Mr. F. P. Nathan (chairman), Christie Cancer Hospital.

Dr. J. Oldfield, Lady Margaret Hospital, Bromley; Mr. Ernest W. Osborn, Stanley Hospital, Liverpool.

Mr. J. B. Parkinson, 10 York Street, Manchester; Dr. H. B. W. Plummer, West Bromwich and District Hospital; Mr. H. M. Phipson, Royal Free Hospital, London; Mr W. Pearson, Gravesend Hospital, Kent; Mr. Geo. Priestman, Bradford Royal Infirmary; Mr. Lawrence Pilkington, Salford Royal Hospital; Mr. W. S. Phophet, Royal Eye Hospital; Mr. A. R. Pollitt, Royal Halifax Infirmary.

Mrs. R. Ramsay, M.D., Glasgow Lock Hospital; Mr. G. Ruddle, Salford Royal Hospital; Mr. T. B. Rhodes, M.B., North Staffordshire Hospital, Hartshill, Stoke-on-Trent; Mr. J. Renshaw, Salford Royal Hospital; Mr. C. S. Risbey, General Hospital, Northampton; Dr. E. S. Reynolds, Manchester Royal Infirmary; Mr. R. Ratcliffe, St. Mary's Hospital, Manchester; Mr. Chas. E. B. Russell and Alderman J. Royle, Ancoats Hospital.

Miss Sparshott, Royal Infirmary, Manchester; Mr. H. D. Stephenson, Gravesend Hospital; Mr. J. E. Smith, Royal Hospital for Children, Bristol; Mr. F. Smith, Warneford Hospital, Leamington; Mr. J. H. Smethurst, Warrington Dispensary; Mr. W. Stevenson, Devonshire Hospital, Buxton; Mr. N. A. Smith, Blackburn Infirmary; Mr. J. Sykes, Royal Infirmary, Huddersfield; Baillie Steel, Glasgow Health Committee; Rev. J. F. Samuel, M.A., Blackburn Infirmary; Mr. E. C. Smith, North Ormesby Hospital, Middlesbrough; Mr. J. H. Shaw, Southport Infirmary; Mr. Arch. Stubbs, Victoria and Albert Infirmary, Winsford; Mr. Fred. Scott, Hospital Sunday Fund, Manchester and Salford; Mr. E. O. Schneider (chairman), Manchester Ear Hospital; Mr. W. Smith-Carington, Christie Cancer Hospital, Manchester; Mr. Fred. Simpson, Manchester Children's Hospital; Mr. Wm. Simms, Manchester Dental Hospital; Mr. J. W. Smith, Manchester Royal Infirmary.

Mr. J. Maxtone Thom, M.D., Royal Infirmary, Glasgow; Mr. A. G. Thomas, M.D., Newport and Monmouth Hospital; Mr. J. W. Taylor, Devonshire Hospital, Buxton; Major C. Tyler, Stockport Infirmary; Mr. Walter Tyzack, Jessop Hospital for Women, Sheffield; Mr. L. Tatham, Royal Eye Hospital, Manchester.

Mr. J. C. Vaudrey (chairman, House Committee), General Hospital, Birmingham.

Mr. J. Wood, Burton-on-Trent Infirmary; Mr. F. Warmsley, Chester Infirmary; Mr. F. H. Westmacott, St. John's Hospital for Ear and Eye; Mr. G. A. Whittaker, Dental Hospital, Manchester; Mr. Chas. T. Walrond, Children's Hospital, Gt. Ormond Street; Mr. Tom Wilson (chairman), Dental Hospital, Manchester; Mr. R. B. Wild, Christie Cancer Hospital, Manchester; Mr. T. C. Waterhouse, Manchester Northern Hospital.

Mr. Chas. Yeomans, Jessop Hospital for Women, Sheffield; Mr. J. Youatt, Royal Eye Hospital.

### Lord Mayor's Welcome.

The Lord Mayor of Manchester (Councillor Behrens), in welcoming the Conference, said: We feel highly honoured that your Association should have selected Manchester for its second annual meeting. Naturally we cannot help thinking that you have come to the place where you may see the latest in hospitals, and possibly this is why you have chosen our city. At no time in the history of hospitals has a congress such as this been more necessary in view of the anticipated changes that will ensue if the National Insurance Bill passes into law. In discussing this question to-day you will have to consider very carefully what the effect is likely to be on the future working and well-being of our hospitals. It appears to me that it might interfere with their voluntary work and with the voluntary support through which they are excellently managed to-day. Whether this would be beneficial or not, it is for you to consider, and I feel sure that your opinions will be carefully weighed by the Chancellor of the Exchequer. (Hear, hear, and applause.)

Sir William Cobbett, chairman of the Manchester Royal Infirmary, was then, as stated, elected to the chair.

Sir William returned the thanks of the Conference to the Lord Mayor for his official welcome, and the Lord Mayor briefly acknowledged it. The Conference then proceeded.

### Criticism of Chancellor's Views.

Discussion of the subject of consideration for the day, "The National Insurance Bill and its probable effects upon the Voluntary Hospitals." was opened from the chair.

Sir William Cobbett: I should like, in the first place, to say a few words about our Association. I am glad to see so large an assemblage of the members of the Association. It shows that the members are taking a keen and active interest in the work of the Association, and probably the reason for that is that they are satisfied that the Association is a body which can be of material help to the provincial hospitals of the country in forwarding their interests, whether in Parliamentary or in other matters. I really do not know how the collective views of the hospitals of the country on the National Insurance Bill could have been placed in a convenient form before the Government—as they were—if it had not been for the existence and action of the Association. (Hear, hear.) On the question how far one may have succeeded in influencing the views of the Chancellor of the Exchequer, or to what extent he may amend the Bill in the interests of the hospitals, I think it would not be safe to say anything. But, at any rate, the Association has been the means of making known to the public the views of the hospitals upon the Bill, and has also been the means of bringing these views forcibly and clearly before the Government. The Association has thereby rendered no mean service to the hospitals of the country, and has thereby shown good reason why those connected with them should join the Association, so that it may increasingly become a large and influential body. (Applause.)

The subject of discussion this morning is the probable effects upon voluntary hospitals of the National Insurance Bill. Speaking individually, it seems to me that it is most advantageous to discuss the Bill with reference to its probable practical effects upon voluntary hospitals. There is no doubt that it embraces a great variety of important subjects, but so far as I am concerned I think it will be best if we confine our consideration of the Bill to these possible practical effects. In the main its effect will, I think, be financial. It has been suggested, I know, that it may make a considerable difference to the amount of work that hospitals have to do. For instance, it has been suggested that it will curtail and circumscribe the work of the out-patient department. But I do not find that medical men are at all agreed on this, and it seems to me it may be safest to assume at present that the work of that department will not be materially affected. As regards in-patients, I have heard it urged that the work of the hospitals will be enormously increased. I am not able to understand the foundation for that argument. I think it is probable that the work of that department will remain very much the same as now, because I cannot help thinking that at the present time we receive and treat as in-patients in the hospitals all those persons who will under the Bill, if it becomes law, hereafter be insured persons. It seems probable that all insured persons will be persons who will be eligible for hospital treatment as in-patients; and I do not understand why the Bill should throw upon the hospitals a very much greater amount of work, as regards in-patients, than they now discharge.

On the other hand, there seems not the slightest reason for supposing that in-patient work will be diminished. And, as we all know, in-patient work is the expensive work of the hospitals. I think, therefore, it may be taken for granted as a general proposition that one thing, at any rate, the Bill will not do—that is, it will not, generally speaking, diminish the work of hospitals and infirmaries. The probability is that they will have just as much work to do as ever and that their expenditure will be as large as ever it has been.

I now turn to the way in which the Bill is likely to affect the finances of hospitals. The Chancellor of the Exchequer has always said that the Bill does not touch hospitals and that they are not affected by it. If he means that the Bill, as it stands, does not affect hospitals, either directly or indirectly, we shall all of us, with all respect, differ from him. An Act of Parliament may have quite as important an effect upon public business and public property indirectly as it has directly. Nay, in some instances it may have a greater indirect effect than it might have by express enactment. When the Chancellor of the Exchequer says he has left the hospitals alone he overlooks the fact that he proposes to tap and divert two of the sources from which voluntary hospitals obtain a very large proportion of their voluntary contributions. In other words, for our allied purpose—a purpose connected with sickness—he intends to tax the working man. He also intends to tax the employer. And working men and their employers are two of the sources on which we have hitherto relied for the voluntary annual income of our hospitals. (Hear, hear.) And when the Chancellor of the Exchequer says that although he is to tax them for a purpose closely akin to hospital purposes he is not thereby touching the hospitals, that surely must be a matter of argument upon which any person is entitled to form his own opinion. And it does not at all follow that the opinion of the Chancellor is the correct opinion, or the opinion that ought to guide public thought in the matter.

It was interesting to observe, when the deputation from this Association was before the Chancellor, what he had to give by way of reasons for his opinion. He said he thought better of human nature than to believe that the men and the masters would cease to subscribe to the hospitals because they had to pay taxation under the National Insurance Bill. I think it must be common knowledge to everyone of us here that when you make a person pay compulsorily towards one or more of several objects all closely allied with other objects of the same character, there is a natural tendency on the part of that person to say, "I am taxed for sickness and I pay for the sickness of people who cannot afford to pay for it themselves, and I am not now to subscribe for an object closely allied to that." That is a view which, it seems to me, is just as much to be expected of human nature as the view which the Chancellor of the Exchequer suggests will be taken by people who subscribe to the hospitals—namely, that they will continue, notwithstanding the taxes, to support the hospitals. Taking as an instance the Workmen's Compensation Act, the Chancellor said that though the Act called upon employers to pay large sums of money for workmen's compensation, the charities did not suffer, though there may have been a slight difference for a year or two. But I do not think that statement is borne out by the facts. The hospitals have suffered in consequence of the Workmen's Compensation Act. At any rate, speaking from my own experience in Manchester, they have suffered. Subscriptions and donations have fallen off, and it has been very much more difficult also to extend the area and the amount of subscriptions and donations.

To sum up his views, as expressed to the deputation from our Association, on the effect of the Bill upon the finances of the hospitals, it comes to this—that the Chancellor of the Exchequer was an optimistic believer for the best. He really had no business argument to suggest to us, to show that the suggestions we made to him were ill-founded and that the belief he professed in the non-injurious effects of the Bill upon the hospitals was a well-founded belief. As opposed to him there is the opinion of practically all hospital experts in the country. Almost without exception they are of opinion that the Bill must necessarily have a serious injurious effect upon the finances of hospitals. On the one hand, the only ground which can be suggested for the belief of the Chancellor that it will not have that effect is an optimistic belief, and on the other hand you have an enormous body of united expert opinion to the contrary. If that be so it is a reasonable thing that hospitals should ask that some precautionary provisions should be inserted in the Bill which shall ensure that they shall not be found to be in a crippled condition two or three years hence, or in a state in which they would be obliged to close their doors and be left to the mercy of the Government in power to make some provision by Act of Parliament for their continued maintenance or resuscitation. (Hear, hear.) Is it satisfactory that the Government should introduce a measure which jeopardises all the hospitals in the country, and makes no precautionary provision to safeguard them from financial difficulty and consequent inefficiency? I suggest that that is not a statesmanlike way of dealing with such an important body of institutions as the hospitals of the country. ("Hear, hear," and applause.) I suggest that to pass an Act of Parliament in the belief that it won't hurt them without having solid business foundation for that belief, and trusting to the possibility that it may be correct, and risking the probability that disaster may ensue, is not the right thing to do—that it renders the Bill as it now stands an imperfect Bill, a Bill which may possibly do a very great amount of mischief, and that unless some remedy is adopted whereby the hospitals may be protected, it would be a misfortune for this country that the Bill should pass Parliament. (Applause.)

As to the nature of the remedy which this Association has suggested to the Chancellor of the Exchequer for the financial difficulties which the Bill is likely to create for voluntary hospitals, our memorial in substance asked the Chancellor to amend the Bill so that the voluntary hospitals should receive a proper payment in respect of each insured in-patient treated within their walls. The suggestion is in the nature of compensation. It is not technically compensation, because of course at present the injury has not been inflicted. All we ask for is that the injury likely to be inflicted upon our finances by the Bill may be met by some countervailing payment. We are possibly within measurable distance of great danger to our finances, and all we have done is to say to the Government,

"Don't place us in that position; insert in your Bill a provision by which, if the difficulty arises, we shall be compensated, and not be brought either to close our doors or to work perhaps half-time." We don't ask for State-aid. It is a misnomer to call such contributions State-aid. We are now getting large subscriptions and donations from employers and workmen, and the probability is that that money will go, under the provisions of the Bill, to the Health Commissioners, and will not come to us. Is it a very unreasonable thing for us to say to the Government, "Out of the aggregate sum you are to get from the fourpences of the workmen and the threepences of the masters, pay us such a sum as, being fairly proportioned to the work we are to do for your insured persons, will form a compensation fund which will protect us from the possibility of financial difficulty and misfortune?"

Sir Henry Burdett pointed out these were not the views of the Hospitals Association, which declined to differentiate where the money was to come from. That was not a question for the hospitals, but for the Government to determine.

The Chairman: I admit that perhaps I am saying more what my own individual views on the subject are than representing the views of the Association. But I was only explaining to the Conference that in making this suggestion to the Chancellor we did not ask him to bind himself to any hard-and-fast rule. We gave him a general indication of the principle upon which we thought a payment might be made to the hospitals. Personally I think there are matters of detail which would have to be taken into consideration.

Mr. Chas. Lupton, chairman and treasurer Leeds General Hospital, discussed the probable effects of the Insurance Bill in the light of the historic work of the voluntary hospitals.

I think the Insurance Bill fraught with such serious consequences to everybody in the country that it is well we should consider it in all its aspects. Personally I have no hesitation in saying that I cordially agree with all that Sir William Cobbett has said in regard to the pecuniary effect of the Bill upon hospitals. I feel it is certain that it will be with very great difficulty that voluntary hospitals will continue to keep up the high level of subscriptions and donations which they have hitherto enjoyed. But I propose to direct my attention to some other effects which the Bill will have upon hospitals. These institutions are the growth of practically a century and a half, and it would be impossible by mere money to replace what has taken so much time to build up. Our country is probably as well supplied with hospitals as any other, and if we have not got quite as many as some other countries, there are hospitals in the United Kingdom to which everybody looks up with esteem and admiration throughout the civilised world. (Hear, hear.) A very high level of practice and treatment characterises the whole of them, large and small. A gentleman who had been spending his time gaining experience in one of the great hospitals abroad—one of the largest in Europe—told me it was a most admirable school for the medical man, but that the patient was not treated as a human being, but only as an object for the advancement of science. That cannot be said of any English, Scotch, or Irish hospital. (Hear, hear.) In addition to the voluntary hospitals, we have also municipal and Poor Law hospitals. But for the purposes of my argument I should like to leave them outside the question. For, though important in themselves, they only do work for the district in which they are situated, whereas in the case of the voluntary hospitals the reason why a sufferer is admitted is because he is sick and in need. The fact that he lives in the next parish, or the next but one, is no reason for preventing him having the advantage of the hospital. (Hear, hear.) This is a characteristic specially marked of the great hospitals of the country—in Manchester, London, Glasgow, and Edinburgh. There we find huge hospitals manned by the best type of medical men in the country, and in each of them there is a great school of medicine attached. In one of these great voluntary hospitals—in Glasgow—Lord Lister carried on his work, and practically revolutionised surgery throughout the whole world. (Hear, hear.) I should like to emphasise the great importance which, in my opinion, is to be attributed to the connection of these great hospitals with their medical schools. In a great municipal hospital, where you may have more beds than you find in our great general hospitals, the people who look after the patients are practically independent persons. They depend no doubt to a certain extent upon a committee whether of the Board of Guardians or of the municipality; but I venture to say that no layman is capable of protecting the patient against irregularities on the part of the doctor. In the great general hospitals the patients are never operated on without a crowd being round most anxious to observe the methods and take all the instruction which the great operators are able to give. The operators are on all occasions working before the power of enlightened criticism. In the same way when the physician goes round his ward he is subject to similar criticism. We provide under this system for the very poorest of our people treatment which a millionaire cannot compass with all his money.

I venture to think that this is a state of affairs which we must maintain. The treatment in these larger hospitals has, in my opinion, a great effect upon the treatment in other hospitals throughout the country. A general system of treatment has been evolved through these great hospitals which has been of the greatest service to our country.

The reason why I say so much about the work of our great voluntary hospitals is that I am greatly afraid that the direct result of the Insurance Bill will be to break up this part of our system. If we are correct in thinking that the effect of the Bill, without some such remedy as Sir William Cobbett has proposed, will be that our voluntary hospitals will lose a great part of their income, the result will be that we shall be turned over in some way to State support. I think the indications are that the Government will try to throw the expense of these hospitals upon the different municipalities, and I want most strongly to urge that we should oppose these larger general hospitals being thrown upon any municipality whatever. (Hear, hear.) At the Leeds Hospital we take in patients from large districts round about, but if in future it is to depend on the city of Leeds for its support the people who regulate its finances will necessarily feel that the hospital must be reserved for those who are taxed to maintain it. The great country districts which depend upon our hospital will thus be entirely excluded from the benefits of its work. There is more than the question of money when it is suggested that these districts should form hospitals of their own. In country districts it would be impossible to find medical men competent to carry on a hospital in the way in which the great city hospitals are carried on. It is not our business to decide how the problem should be met. What I wish to point out is that there is a very grave problem involved beyond the mere question of supplying us with so much money, and I for one would regard it as a retrograde step if hospitals which serve the country were to be degraded into hospitals serving towns or sections of the country.

### Hospitals and Insured Cases.

Dr. D. J. Mackintosh, M.V.O., Western Infirmary, Glasgow (one of the hon. secretaries of the Association), read a paper on the subject.

It has been said (Dr. Mackintosh remarked) that the principles underlying the National Insurance Bill are to purchase by a great, united, and national effort, for our vast working population, efficient medical attendance when sickness comes, proper medicine, suitable surgical appliances, and proper nursing. How is this to be accomplished? The Bill makes provision for domiciliary treatment, but no provision whatever is made for institutional treatment beyond providing sanatoria for the treatment of tuberculous cases. That being so, it is evident that when an insured person requires a serious surgical operation, or requires treatment by a specialist and skilled nursing, he must seek admission to a voluntary hospital. Is it reasonable that a Bill should be presented to Parliament which does not make provision for carrying it out in all its details? Can the Bill be amended in such a way as to permit of the voluntary hospitals being maintained in their present state of efficiency without becoming State institutions? I am not alone in the belief that if the Bill is passed in its present form the voluntary hospitals will suffer so seriously as to render them inefficient, both as curative institutions and teaching schools. If the impression goes abroad that the voluntary hospitals may soon be taken over by the State, those who have it in their hearts to leave legacies to these institutions will hesitate, unless some authoritative pronouncement is made now which will dispel such an impression. Who ever heard of anyone leaving a legacy to a parochial or State-supported hospital?

But instead of having mischievous results, the National Insurance Bill might be so amended as to prove a tower of strength to the voluntary principle. It might also have the effect of preventing any abuse which may at present exist in the working out of the voluntary system. This can be brought about, first, by the Insurance Bill being so amended as to prevent anyone gaining admission to a voluntary hospital whose income is over the insurance limit; and, second, by making provision in the Bill for the Insurance Commissioners defraying the cost of treatment of each person admitted to the hospital.

The question of remuneration of the medical staff is a matter which, in my opinion, can be adjusted to the satisfaction of the staffs of voluntary hospitals. If the medical staff is to be paid by the hospital the question of their remuneration as medical officers to the institution must be considered from a broader standpoint than the mere pecuniary return which they might receive as medical officers. Take, for instance, the case of a teaching school, where the senior surgeons and physicians receive in cash the whole of their clinical fees, amounting in some cases to over £300 per annum, plus the honorarium of £50. This cannot be taken as a standard for every voluntary hospital. Personally, I hold the opinion that every man should be paid for his services, either directly or indirectly; but it must be evident to everyone that no final decision can be arrived at on this point until we really know whether the State proposes to make all hospitals State institutions, or whether the present voluntary system is to be continued and developed.

It is in my opinion, a most dangerous course for hospital managers and administrators to lie low and do nothing for the present, but to wait and see. We must try by every possible means in our power to persuade the Government to make such amendments in the Bill as will not only safeguard the voluntary hospitals but render them more and more efficient, and at the same time less liable to abuse; or let them declare at once that the voluntary hospitals are no longer required, that they have outlived their usefulness, and that the State is prepared to take them over. There is no middle course. If the Bill becomes law in its present form the voluntary hospital will very soon cease to exist. Why should gentlemen who are at the head of large businesses, and professional men with large practices, devote their time and attention to the management and working of the voluntary hospitals if they are no longer required?

The Chancellor of the Exchequer came very near the point when he suggested to the deputation which met him that the remedy for any hospital abuse was very largely in their own hands. He pointed out that if the approved Societies and Health Committees did not contribute a sufficient sum to retain the efficiency of the hospitals, he considered that the managers of voluntary hospitals were quite entitled to ask any applicant for admission the simple question, "Are you insured?" If they are insured they have no right to be admitted to the hospitals without the hospitals receiving an adequate sum for their treatment. And if unsuitable patients are sent by doctors who are paid by the Insurance Commissioners, they also should be told that the hospitals will not take the burden off their shoulders. So long as the voluntary system continues the voluntary hospitals will be in a position to say to the Insurance Commissioners, "We cannot take patients unless you are prepared to meet us; we are free contracting parties." Nothing will be satisfactory until the Bill is so amended as to make it compulsory for the Insurance Commissioners, not the approved Societies and Health Committees, to contribute to the hospitals a sufficient sum to recoup them, not only for the hospital outlay, but for the payment of the staff, bearing in mind that they are really in receipt of funds both from the employer and the employé to overtake this work.

As to the financial prospects of the voluntary hospitals under the Bill as it stands, no one with a knowledge of the working of the voluntary system would compare the results of the Employers' Liability Act on hospital subscriptions with the certain consequence of the National Insurance Bill as at present framed. No one took such an extreme view of the Employers' Liability Act as to say that it would seriously affect hospital subscriptions from employers and employés. But if the same policy is to be driven home in a more exaggerated form by the passing of the Insurance Bill, there can be no doubt about the ultimate effect of such legislation. I would prefer to have the Bill amended in such a way as would make it possible for anyone to receive treatment in a voluntary hospital who is above the pauper class and is not in a position to pay for efficient treatment outside. This would encourage benefactors to increase their gifts rather than withhold them, as they will have the full assurance that the Bill will protect the hospitals from abuse. In no way could this be carried out more effectively than by a system of Government inspection. No institutional treatment is provided in the Bill for the working-man with a small income who is unable to pay the fees of a nursing home and a specialist when he suffers from an ailment which it is impossible to treat efficiently in his own home. There is no place for him unless he enters a voluntary hospital, and why should any Bill be passed which would tend to withdraw a single penny from the funds of the voluntary hospitals?

I would like to refer in a word to the probable effect of the Bill on the working of the out-patient departments of our voluntary hospitals. With the exception of accidents and urgent cases, the out-patient department of every voluntary hospital should be restricted to the treatment of patients who are not in a position to pay for adequate treatment outside, and who are, from a hospital point of view, suitable for treatment in a charitable institution. I would suggest that when a patient has been seen and ordered treatment which can only be efficiently carried out in an institution, by either a School Board medical officer, an insurance doctor, or anyone paid by the Poor Law or the State, the hospital should not treat those cases gratuitously.

The medical men who will serve under the insurance scheme are not supposed to undertake work which can only be done in hospital, and while the out-patient department will be relieved of a number of trivial cases, the number of cases seeking admission to the wards will tend to increase rather than decrease. And unless the Bill is amended so as to secure the necessary funds for the upkeep of hospitals, no scheme of national insurance will be complete or satisfactory until proper facilities are provided for hospital treatment. It must be borne in mind also that the Bill makes no hospital provision for the workman's wife and children, with the exception of the 30s. under the Maternity benefit.

Hospital managers should leave no stone unturned until the question of hospital treatment for the poor above the pauper class receives more consideration from the Government and the House of Commons. We have come to a crisis in the history of the voluntary principle. The increasing burden which is rapidly being thrust on the finances of all our voluntary institutions is a most serious matter, and I fear a National Insurance Bill, instead of relieving this increasing burden, if passed in its present form, will tend to increase it materially. Why should we not co-operate with the British Medical Association and formulate a scheme which would demonstrate clearly to the member of Parliament for every district where there is a voluntary hospital, that unless he can submit to his constituents an incontrovertible argument against the continuation of the voluntary principle he should pledge himself to support any reasonable amendments which will not only maintain the voluntary hospitals in their present state of efficiency, but keep them all up to date as the centres of the most advanced methods of medical treatment and research?

### Mr. Howard J. Collins, House Governor, Birmingham General Hospital, read a paper in which hospital treatment as a "benefit" was considered.

It is difficult to conceive, he said, of an Act of Parliament for the insurance of workers, as a national provision for the health of the working classes, which provided only for such illness as could be treated by domiciliary visits of medical practitioners. There is much talk about the Act, and such terms as "State aid" and "State control" are bandied about, but no one appears to want State aid or State control—least of all the State as represented by the Government. Certainly hospital managers do not desire it if it means the reducing of voluntary hospital work to the level of the Poor Law infirmary. On the other hand, no responsible manager or officer of a voluntary hospital can have the slightest objection to inspection.

It seems to me clear that voluntary hospitals as such are not concerned in the arrangements made, or to be made with medical practitioners. But as this part of the Act only provides for the treatment of the minor conditions, there must be considered the serious operation cases, as well as the medical and surgical cases requiring thorough investigation and skilled treatment, operations and nursing. If it is intended to include in the provision for tubercular disease all treatment, including operative work, for tubercle of joints and other conditions, it would enormously relieve the present pressure on voluntary hospitals. But in this case "Sanatoria" seems to be a misnomer. I should like to clear away the misconception that voluntary hospitals want more cases. I believe it to be a fact that most hospitals are already overwhelmed, and would welcome any reduction which relieved the pressure and so allowed more attention to be given to the really serious cases.

If the Act passes in anything like its present form there must be a material alteration in the practice of voluntary hospitals. So far as the out-patient department is concerned, I believe that the Act will enable hospitals to refer insured persons to the doctors provided for them, but undoubtedly there will be cases referred to the hospital. Hospital work must continue, not only in the treatment of serious medical and surgical conditions, but in the training of medical men and of nurses and scientific experts, in the investigation of disease, in the study of special diseases, and the publication of reports, as well as the immediate treatment—the saving of lives and of limbs—in cases of accident. All this has been the work of voluntary hospitals for many years, and in my opinion it should continue to be so. We do not, therefore, oppose the Bill, but ask for its extension, so as to include these serious conditions, which will undoubtedly occur to insured persons. It seems to me that it is not for us to suggest how this should be provided for, but to point out the absolute necessity of some portion of the money contributed by employers and employed being available for the treatment of the employed when seriously ill—in other words, a "benefit" under the Act.

I have heard suggestions made:

1. Of a grant in aid in proportion to the amount contributed locally, as in the case of Universities.
2. A grant in aid according to the number of cases treated.
3. A proportionate grant calculated on the amount of work, with or without a deduction for the income received from investments and legacies.
4. A proportionate amount for such cases as are not otherwise provided for.
5. A definite payment for each insured person.
6. A definite payment for the actual cost of each patient, according to the length of stay.

But personally I am content to leave this matter to the Chancellor of the Exchequer and his advisers or to the Insurance Commissioners when appointed, provided that the Bill sets apart funds for a "benefit." Otherwise the voluntary hospitals must inevitably dwindle, and the question then remains whether they should be allowed to be starved out so that the most carefully and economically managed institutions would last longest, or should be—as I believe many members of the medical profession think—handed over to the Health Authorities under the Act for them to maintain. In either case there must be a period, long or short, for dwindling, both in quantity and quality of their work. Personally, I feel strongly that hospital treatment should be made a "benefit" under the Act, and that if this is carried out voluntary hospitals will continue to do the valued work which they have done in the past, and probably with even greater success, because of their being relieved of the minor cases which will then be provided for otherwise.

### Mr. J. Courtney Buchanan, of Lincoln's Inn, Barrister-at-Law, Secretary of the Metropolitan

Hospital, London, read his paper on the Voluntary Hospitals and the National Insurance Bill.

I submit that there is little analogy between this Insurance Bill of 1911 and the Employers' Liability Act of 1880 and the Workmen's Compensation Act of 1906. The National Insurance Bill is a measure of much larger scope; its effect will be much greater, more direct, and not only immediate, but also continuous; and, when once the Bill has become law, it will be of no avail for the voluntary hospitals then to object to any work which may be thrust upon them.

Now, regarding the position not quite so much in relation to the hospitals' work alone, but keeping in sight at the same time the position of other agencies which minister to the sick poor, I suggest that the National Insurance Bill may provide the necessity, or the means—at present it is impossible to say whether directly or indirectly—of changing the whole medical service of the community, with which, as you are well aware, there is, and has been for some long time, considerable dissatisfaction. The voluntary hospitals are only a part, but still by far the most important part, of our medical service.

If the National Insurance Bill is to achieve anything like the good which is hoped from it, the poor must have the relief they require in the *early* stages of their diseases, and care must be taken against relief being delayed until disease is so far advanced that health is permanently impaired and remedy impossible. With this secured, the Bill would be an effective instrument for improving the general health of the community, and much sickness would be altogether prevented.

Having regard to the changes which must sooner or later follow consideration of the reports of the recent Royal Commission on the Poor Laws and Relief of Distress, I trust that a scheme will be worked out which will not only secure to the hospitals immunity from undue interference and adequate payment from State funds for work done for the State, but also define the function which the voluntary hospitals should fulfil in relation to other agencies, whether State, or municipal, or voluntary, which deal with the medical relief of the sick poor.

We need no longer treat the trivial ailments of insured persons, and it would seem that insured persons should only attend at the hospitals on recommendation by their own doctors. This will mean that, for insured persons at all events, the out-patient department will become more and more what, perhaps, it should be for *all* persons—namely, a consultative department for serious cases.

It may be that the Local Health Committees, whose work will be under the supervision of the Insurance Commissioners, will help the hospitals to obtain proper return for work which they do for insured persons; but it has been our experience hitherto that such approved societies of working men sometimes demand privileges and authority out of all proportion to their contributions. This, however, may be corrected if the hospitals are able to secure representation upon the Local Health Committees.

In the course of their insurance work the Local Health Committees may become powerful agencies for organising, registering, and co-ordinating the whole of the assistance available for the sick poor, so that not only will patients be able to obtain the relief they require quickly and readily, but also it will be possible to have patients watched by district nurses after they leave the hospitals or other places where they have been medically treated. This will lead, I hope, to the full development of the constructive or positive work of our almoners' departments, because, if fully developed and adequately staffed, the almoners' departments will be, in my opinion, of great service to the Local Health Committees.

The Insurance Bill may be the means not only of improving the general medical service of the community, for general practitioners would thus be in constant touch with the hospitals, which are, in fact, schools of medical learning, but at the same time, by co-ordination and co-operation, it may help to produce friendlier relations between the various members of the several medical services and the hospitals.

If, then, the patients are not allowed to go to the hospitals as before, some definite arrangements must be made for them. I recommend that insurance centres should be distributed over the several areas under the jurisdiction of each Local Health Committee; and I would suggest for their working the principles laid down by the majority of the Poor Law Commissioners for their provident dispensaries.

In answer to critics who hold that State aid means State control, I would say that hospitals have nothing to fear from inspection. We rather welcome it, *always provided that* inspection and control are conducted by a trained and experienced body, and that hospital officers are not harassed by the interference of those who have no practical knowledge of hospital administration. For London, at all events, I venture to say, if I may do so without impertinence, that the King's Fund, though not representative of all the hospitals, already and obviously fulfils these purposes; and, at times such as the present, if made representative of all the hospitals under its control, could not the Fund serve as a central body to advise the Government, and explain after full discussion by constituent hospital authorities the educated views of hospital workers? The hospitals of London work under quite different conditions from those in the country, and the report of the special Out-patient Inquiry Committee will, no doubt, contain suggestions for the solution of our present difficulties in London; and some uniform method of admission may be determined. If the hospitals would unite, or if this Association would combine with the British Medical Association (who have done the bulk of the work on medical questions involved in the National Insurance Bill), I cannot but think that a sound scheme for the future working of the hospitals might be evolved.

The chief object to which I fancy the National Insurance Bill is directed is the improvement of the health of the community at large, and in this connection the provision of assistance to the sick poor in the early stages of their diseases is, I take it, a matter of the greatest importance. I have suggested a means by which in my judgment early application for assistance can be facilitated.

This suggestion of mine has a further significance in that it will bring the hospitals into closer touch with other agencies for treatment of the sick and, through the Bill, under the immediate notice of the State, which should be to the hospitals' material advantage.

The National Insurance Bill may be the means of :

(*a*) Preventing overlapping, which is wasteful; and

(*b*) Making other provision for the treatment of patients who are either not qualified for hospital treatment, or who could be just as well treated elsewhere.

Hospitals will inevitably be hard hit as far as their finances go by the Bill, and the extent of their loss may be very serious indeed.

However, it may be that the reforms or alterations which will be hastened by and result from the operation of the Bill will be the means, not only of correcting the overlapping and the treating of patients who, for one reason or another, ought not to be treated, but it may also make up to the hospitals the loss of income with which we are told they are threatened.

Therefore, I have suggested that the British Hospitals' Association should combine with the British Medical Association as two of the best informed and most representative bodies, in order to draw up a scheme which will define the work which the hospitals could do, and would do, best in relation to other agencies, and thus assist the Government to carry out their desire and intention, repeatedly and forcibly expressed, of maintaining the voluntary system.

### The Discussion on the Papers.

Mr. James Smith, Bristol Children's Hospital:

We all seem to fear the effect of the National Insurance Bill on the existence of our hospitals. Several speakers have made reference to State-aid as though it were a bogey and very much to be dreaded. I don't think there is any necessity to conclude that hospitals will be taken over by the State just yet. There is a via media. Instead ot hospitals being wholly State-supported they may be State-aided. We have not to go far to find a parallel. There was a time when all primary schools were voluntary schools, but seven or eight years ago the time came when Imperial Parliament made a grant in aid of the schools. I think we have now arrived at that point with regard to the hospitals. No doubt in time hospitals will be in the same position as schools—entirely supported by the State. We seem to be rather shy about that. I do not think the majority of the Conference agree with some remarks made this morning on the subject of State-aid. I think the time has arrived when we should go to the Government and say that we think the time has come for State-aid. I speak especially on behalf of children's hospitals which, because they are small, seem to me to receive a smaller amount of attention and support than they deserve.

Sir Henry Burdett spoke on the Bill and its consequences:

I am strongly in favour of the Insurance Bill. I have spent nearly all my leisure for 45 years in work which I hope has assisted the improvement and well-being of the people of the humbler classes, and I do think it is time we had a National Health Insurance Bill passed which should play an important part in encouraging thrift and self-help among all classes of the community. (Hear, hear.) I wish to make that perfectly clear because I am certain it is of the first importance that Mr. Lloyd George should understand that the responsible hospital managers of this country are absolutely with him in endeavouring to lessen the burden and the strain, and the misery and loss, of illness and sickness and accident to the poorer members of our race. (Applause.) But when we have said that we have to remember that Mr. Lloyd George has tackled probably the most intricate and difficult problem which any modern statesman can possibly have to handle. He has tackled it in a masterly way, for those who study the Bill must realise that it is a great conception. But it has been done too hurriedly, and what the Bill lacks is time—time for its defects to be provided for. It would be a misfortune to hurry a great and complicated Bill like this through Parliament, if it resulted—as it certainly will result if my knowledge of hospital finance is worth anything—in the death of the voluntary hospital. Speaking as one who has investigated all hospital systems and inspected hospitals of every type, I would say that if we are to have the abolition of the voluntary system of hospitals in this country—that great system which is the admiration of the whole world—such abolition would be a grave peril. This system makes it a positive fact that the lot of the poor man in the great voluntary hospitals of the country is better than money can provide for the richest member of our community. Under this system the principle kept first and always in view is that the patient and his welfare must be first considered, and not science. Patients are not mere "cases": they are men and women. (Hear, hear.) It is because our voluntary hospitals give us this advantage—this recognition of the claim of the humblest in their day of sickness to be regarded as human beings, and to be cared for and attended as if they were the richest among us—I say, because of this, it would be a bitter wrong to substitute State-aided hospitals and abolish the voluntary system. (Hear, hear.)

Take the Poor Law infirmary—and we have some splendid examples of them in this country—if you are a ratepayer you can only go into the infirmary to see it once a month, and that on a day which is fixed, and at certain hours. In the case of the voluntary hospital you can go in and see the place and everything that goes on under the gaze and inspection of the people. In State-aided hospitals freedom of independent inquiry is largely absent. It must necessarily be absent. And you find that the patients become numbers and "cases" rather than individuals. That tendency, indeed, steadily increases all over the world. I hope, therefore, we shall never have State-aided hospitals in lieu of voluntary hospitals. But as I may be put down as a mere enthusiast, I will come down therefore to the matter from the business point of view. What are the economic considerations with regard to State hospitals? Hospitals require in this country from £10,000,000 to £15,000,000 a year. This is equal to more than one-sixth of the national revenue ten years ago, or considerably more than 25 per cent. of the whole revenue raised, according to the latest returns, in 1905-06 from local rates, including Poor Law, county and municipal authorities, sanitary authorities, and elementary education authorities. In other words, the rate per head of the population, if we are to have State hospitals, would be increased from £1 14s. 1d. to over £2 2s. The probable effect on the hospitals of the Insurance Bill is that at least one-half of the whole revenue received by the voluntary hospitals must be adversely affected by the Bill. You know that some voluntary hospitals get by far the larger portion of their revenue—a few get nearly the whole of it—from the contributions of artisans and their employers. And I make bold to say that if this Bill passes it is perfectly certain that those contributions must be materially diminished, if they do not disappear altogether. I am not speaking on theory, but as one who has been going through the great hospitals of the northern counties where the system of workmen's subscriptions prevails. The unanimous opinion there is that the Bill means probably that this source of income for the voluntary hospitals from being a mainstay may become a very, very small thing indeed.

The solution of the question, in my view, is for Clause XV. of the Bill, which provides for sanatoria benefit, to provide also for hospital benefit. (Hear, hear.) I would like to get an expression of opinion of this Conference that we are unanimous in standing by the voluntary principle, and that we cannot accept doles or State-aid, but that what we will do is to conduct our hospitals on a business basis, and that, if the State ask us, as it will ask us under this Bill, to provide accommodation—especially in-patient accommodation—for the people insured by the Bill, the State will be prepared to pay us pro rata the cost of these cases, and at the same time add something for remuneration of the medical men. (Hear, hear.) We request and must absolutely insist upon the amendment of Clause XV. to provide for hospital benefit. If this is done I make bold to say—and I am speaking with some knowledge of the inner working of the position of the Bill at the moment—that you may take it for granted that such a resolution will be of the first importance and may have a most momentous effect.

Dr. T. B. Rhodes, North Staffordshire Hospital:

Honoraries and Insured Cases.

I hold a somewhat peculiar position, in which I have an opportunity of seeing both sides, and I can assure you that where general practitioners are the honoraries of your hospitals payment for insured persons means payment for the doctors and eventually State control. My own hospital is having a special meeting of the Committee to decide whether they will not on their own account memorialise the Chancellor of the Exchequer—they have felt so strongly in regard to this question of the payment for treatment. In the memorial presented by this Association "cost actually incurred" is what is mentioned, and it may be said that that does not mean treatment by the medical man. But it is bound to follow. Let A and B be two medical practitioners in a certain portion of a town. A is on the staff, and he happens to be on duty on a particular day as the surgeon. B is not. The two practitioners are in competition. B has a case for which he is paid a certain sum a year by the State under the Insurance Bill. An urgent operation is required for appendicitis. He takes the case to the hospital. The honorary must come immediately to do it for nothing, although he may have a very important engagement. In any case he has to come and operate on this case for which the other man is getting paid. And he does it for nothing. Another point is this: If you pay the doctor, A and B are still the same. B will say, "Why should not I do the operation and get the two guineas?" (*A Voice*: Because he cannot.) I do not say he could do it, but I guarantee that 20 per cent. of the general practitioners who are not on the staff in the district I come from would ask to do it.

Mr. J. A. Cowley, Secretary Victoria Infirmary, Northwich:

I speak for a hospital of twenty-five beds, and the position stated by the last speaker is accentuated in our case. The whole work is done by the general practitioners in the district without payment. Even if the suggestion of the Hospitals Association is adopted in the Insurance Bill, we shall still have a difficulty if the general practitioners are to do this work. If a certain number of them are to be paid by the State—if a hospital is to receive State-aid, then these men will want payment for the work they do and the expenditure of the hospital will necessarily increase. I hope the Bill may be amended so that it may be acceptable to all the various interests which it is bound to affect.

The discussion for the day closed at this point. Members of the Association were entertained at luncheon in the Midland Hotel by invitation of the Boards of Management of the United Hospitals of Manchester and Salford. In the afternoon a visit was paid to the Manchester Ship Canal, where a steamer was placed at the disposal of the Association by the directors, and members were conveyed to Barton Bridge. In the evening a reception was held at the Town Hall by the Lord Mayor and Lady Mayoress of Manchester.

Resolution on the Insurance Bill.

On the resumption of the Conference on Friday, September 29, again under the presidency of Sir William Cobbett, formal business—the election of officers and council for the ensuing year, etc.—(as given at the close of our report), was taken.

Thereafter the debate on the National Insurance Bill was resumed on a proposition brought forward by Dr. Mackintosh, M.V.O., Glasgow, who said he had received a letter from a member of the Association who had been unable to remain for the second day's proceedings, and who suggested that the views of the Conference on the Insurance Bill should be focussed in some way. The member sent a resolution with his letter and said that had opportunity offered he would have been prepared to move it.

The resolution in question was as follows:—

**Text of the Resolution.**

"That this Association of hospital managers and administrators is convinced that the National Insurance Bill, if passed in its present form, will seriously prejudice voluntary hospitals all over the country, not only financially, but in their relation to the medical profession, and unites in calling on the Government so to amend the Bill that the continued existence of voluntary hospitals may be safeguarded financially, and their efficiency as curative institutions and schools of medicine may be maintained and developed."

Dr. Mackintosh: I formally move the resolution.

Sir Henry Burdett: I have much pleasure in seconding.

The resolution comes before us because it is felt by the Conference—and a large number of friends not here this morning have spoken to me about it—that it would never do, after the discussion we had yesterday, to dissolve without placing on record our considered and I hope unanimous opinion. (Hear, hear.) It has to be remembered that the effect of the Bill upon the number of patients in hospitals has yet to be discovered. All we know is that, accordng to the closest estmates of some of the best authorities, 10,000,000 persons will become insured in all probability under this Bill, and that a large number of them will want hospital care. Now, the voluntary hospital was established for the relief of the bread-winner, who, when well, can maintain his family and himself comfortably and without outside aid, but who cannot provide in addition the cost of hospital care and medical treatment for himself and his family. (Hear, hear.) That is the bed-rock on which the voluntary hospital rests. (Hear, hear.) The Poor Law provides for all the cases below those covered by this definition, and it is our grand privilege as voluntary hospitals to do the work of the Good Samaritan and help the man who helps himself when misfortune comes upon him by the act of God. That is the principle which has made the work of the voluntary hospitals such a wonderful work to engage in by knitting us together and making everyone of us better men and women. There is no doubt that the best voluntary hospital stands out as the very highest unit of efficiency known in hospital matters. The ten millions of insured persons under the Insurance Bill will become ineligible for hospital treatment in the voluntary hospital on the basis and foundation of its work. That is a matter which I believe the Chancellor of the Exchequer has not quite understood or recognised. That is why I said yesterday that through this Bill we are going to have created an army of new applicants for hospital relief in the voluntary hospitals. That army will be made by the act of the Government, and we ask that this Bill, which must seriously affect our revenue because it aims at the sources from which half of it has come, must provide for the hospital benefit of the insured as well as for their sanatoria benefit. We want to have a modification made in Clause XV. which will confer that power on the Insurance Commissioners. Then the Government can come to the voluntary hospitals and ask them to meet them by providing the hospital care which the insured will need. In that way I believe we shall ultimately welcome the Bill, because it will bring about a re-organisation of our whole present system of hospital relief and ensure that what has been known in the past as in-patient abuse will be largely if not entirely removed. We have also to remember the fact that if Clause XV. is altered in order to give power to confer hospital benefit it may get the Government out of a great difficulty. Sanatoria benefit is not a cure for tuberculosis. It is only a palliative, and many people think that the sanatorium will be very useful as a home for the dying—for cases of tuberculosis in their latter stage. I know and I agree that medical science recognises that sanatoria care, being only a palliative, must be something which cannot endure. The time it may endure is not known, but it is perfectly certain that it will ultimately—and I hope speedily—be replaced by scientific developments which will finally provide a cure for, or for the extermination of, tuberculosis. Thus, if great care is not taken at the commencement, very large sums of money may be sunk in buildings for sanatoria benefit which, in twenty years' time, may be standing unoccupied as monuments of hurried legislation, in the same way as certain forts are still standing in England to remind us of the panic of invasion that spread in Lord Palmerston's time. In asking for this protection for hospitals by providing for hospital benefit in the Bill, I believe we are doing a very great administrative service for the success of the measure. For there must be so much less money for sanatoria benefit if an adequate amount is provided for hospital benefit as well.

Well, the Association raises its flag in this matter. The members go back to their work in various parts of the country, and the Council will have to consider how our organisation can be perfected. The way to proceed, I think, is for every voluntary hospital in the country to get together its supporters and make it clear to the Parliamentary representatives of their district that if this Bill passes unaltered, and as it stands to-day, it must mean the death of the voluntary hospital. I hope local and district committees will be organised to represent all the voluntary hospitals in each district, and that the Council will get the names of those who will act for them in commencing the organisation in such districts. Our effort must be made to organise in pursuit of the policy declared in our resolution, so that we may secure that the voluntary hospital shall not be injured under this Bill, but that it shall go forward, as I hope, with brighter prospects and enabled to render even greater benefits to the community in the future than it has rendered in the past. By organisation a way will be found to free us from the financial dangers by which we are threatened.

Mr. W. G. Black, Glasgow Royal Infirmary and Glasgow Eye Infirmary:

Some Glasgow Figures.

So far as Glasgow and the North of England are concerned, our hospitals are in an entirely different position from the hospitals of London. I will give one or two illustrations of how we are likely to be effected by the Bill. Taking the two infirmaries with which I am specially connected, there is the case of the Glasgow Eye Infirmary, an old institution. Last year the total number of cases under the medical officers was 28,681. I have analysed the income of this institution for the last three years. I leave out of account legacies, income from invested funds, and everything except the ordinary annual subscriptions. I divide these into two portions, and the result is this: In 1908 we drew £1,090 from ordinary subscribers, and from the employés in the great public works of Glasgow we drew £2,272—more than double the other amount. In 1909 we received from ordinary subscribers £1,052 and from employés in public works £2,112; and in 1910 the figures were £1,060 and £2,133 respectively. In the Eye Hospital of Glasgow we value highly the subscriptions from the working classes. They are mainly derived from small voluntary deductions from their wages, and a sum in each works is divided by the vote of the men. If the Insurance Bill is passed in anything like its present form we cannot expect that source of revenue to continue. And the ordinary subscriptions, apart from those of the workmen, are very largely made up of contributions from their masters, who go hand in hand with them in maintaining the hospitals. We therefore, at this particular infirmary, look forward with the very gravest feelings if this Bill is to pass in anything like its present shape. You cannot expect the working-man to allow a deduction from his pay for voluntary hospitals and another deduction for sickness insurance under the Bill; and in the same way you cannot expect masters to give voluntarily and at the same time under the Bill to two similar objects. In the case of the Glasgow Royal Infirmary the employés' subscriptions were £8,000, as against £11,000 of ordinary subscriptions, and in 1910 the figures were £8,222, as compared with £11,000 of ordinary subscriptions. In the last three years we have received from the working-men of Glasgow for the Royal Infirmary there a sum of £24,500, as compared with a total of ordinary subscriptions of £35,666. Under the Bill these sources of revenue will to a large extent be dried up, and if they are dried up what is to become of our voluntary hospitals?

The Chairman put the resolution, and it was carried unanimously amid loud applause.

